# Molecular Fingerprint of High Fat Diet Induced Urinary Bladder Metabolic Dysfunction in a Rat Model

**DOI:** 10.1371/journal.pone.0066636

**Published:** 2013-06-24

**Authors:** Andreas Oberbach, Nico Jehmlich, Nadine Schlichting, Marco Heinrich, Stefanie Lehmann, Henry Wirth, Holger Till, Jens-Uwe Stolzenburg, Uwe Völker, Volker Adams, Jochen Neuhaus

**Affiliations:** 1 Department of Cardiac Surgery, University of Leipzig, Heart Center Leipzig, Leipzig, Germany; 2 Integrated Research and Treatment Center (IFB) Adiposity Diseases, University of Leipzig, Leipzig, Germany; 3 Department of Functional Genomics, Ernst-Moritz-Arndt-University Greifswald, Interfaculty Institute of Genetics and Functional Genomics, Greifswald, Germany; 4 Department of Pediatric Surgery, University of Leipzig, Leipzig, Germany; 5 Interdisciplinary Centre for Bioinformatics, University of Leipzig, Leipzig, Germany; 6 Department of Pediatric Surgery, Medical School, University of Graz, Graz, Austria; 7 Department of Urology, University of Leipzig, Leipzig, Germany; 8 Department of Cardiology, University of Leipzig, Heart Center Leipzig, Leipzig, Germany; University of Cordoba, Spain

## Abstract

**Aims/hypothesis:**

Diabetic voiding dysfunction has been reported in epidemiological dimension of individuals with diabetes mellitus. Animal models might provide new insights into the molecular mechanisms of this dysfunction to facilitate early diagnosis and to identify new drug targets for therapeutic interventions.

**Methods:**

Thirty male Sprague-Dawley rats received either chow or high-fat diet for eleven weeks. Proteomic alterations were comparatively monitored in both groups to discover a molecular fingerprinting of the urinary bladder remodelling/dysfunction. Results were validated by ELISA, Western blotting and immunohistology.

**Results:**

In the proteome analysis 383 proteins were identified and canonical pathway analysis revealed a significant up-regulation of acute phase reaction, hypoxia, glycolysis, β-oxidation, and proteins related to mitochondrial dysfunction in high-fat diet rats. In contrast, calcium signalling, cytoskeletal proteins, calpain, 14-3-3η and eNOS signalling were down-regulated in this group. Interestingly, we found increased ubiquitin proteasome activity in the high-fat diet group that might explain the significant down-regulation of eNOS, 14-3-3η and calpain.

**Conclusions/interpretation:**

Thus, high-fat diet is sufficient to induce significant remodelling of the urinary bladder and alterations of the molecular fingerprint. Our findings give new insights into obesity related bladder dysfunction and identified proteins that may indicate novel pathophysiological mechanisms and therefore constitute new drug targets.

## Introduction

Metabolic syndrome and diabetes mellitus are common in highly industrialized countries with Western lifestyles [1]. Interestingly, voiding dysfunction is reported in 80% of individuals with type 2 diabetes [2]. Type 2 diabetes is a well-known comorbidity of obesity and several studies support the relation between voiding dysfunction and diabetes [3]–[5]. A number of debilitating urological symptoms attend obesity and/or diabetes related bladder dysfunction including impaired bladder sensation, impaired detrusor contractility, and increased bladder capacity leading to significant distress, limitations in daily functioning, and poor quality of life [6]–[10]. Recent literature showed that the classic symptoms of hesitancy (62%), reduced stream (52%), and incomplete emptying (45%) were prevalent. The symptoms of nocturia (87%) and urinary frequency (78%) were the most common in diabetic patients [11]. The diabetic bladder dysfunction is thought to be a stepwise process with storage problems and an overactive bladder in the early phase, which gradually turns into an atonic bladder with voiding problems in the late phase [12].

Those pathological bladder conditions represent distinct clinical entities, implying distinct pathomechanisms. Obesity itself is a very complex phenotype and might involve a wide spectrum of metabolic changes, such as disturbed glucose homeostasis, chronic inflammation, and disturbed lipid metabolism [13]. In our previous studies we focused on cell culture experiments to examine the impact of lipid metabolism on bladder cells [14], [15]. In response to elevated palmitate we found significant alterations of cell vitality and increased expression of inflammatory proteins, such as MCP-1 [15]. Interestingly, MCP-1 is not solely a potent chemoattractant of macrophages supporting tissue inflammation but was also described as a major regulatory protein leading to insulin resistance [16]. Those results demonstrated direct link between fatty acid metabolism and bladder cell dysfunction in vitro. Therefore, in the present study we examined the effect of high fat diet on bladder metabolism in vivo. To reflect our previous in vitro findings we chose a HFD induced rat model with especially high palmitate (C16∶0) concentration of 11.4 fold compared to chow diet. We primarily focused on metabolic alterations in the bladder tissue of obesity rat model. Moreover, the pathophysiology of obesity related bladder dysfunctions is multifactorial, including neuronal, urothelial, and detrusor smooth muscle alterations [7]. Therefore, general alterations in metabolic conditions may be a key event in the induction of bladder dysfunction.

Urinary bladder dysfunction has been studied in rats with metabolic syndrome previously [17]–[19]. Interestingly, obesity but not diabetes alone impaired voiding function in female rats. Voiding dysfunction such as voiding frequency, residual volume and contraction pressure were significantly influenced by obesity phenotype independently of glucose homeostasis in this rat model [4]. It is still unclear which metabolic alterations related to diet induced obesity are responsible for these pathophysiological conditions. A 2D-DIGE study of streptozotocin (STZ)-induced proteome alterations in diabetic rats revealed that the development of diabetes related complications involves down-regulation of structural and extracellular matrix proteins in bladder smooth muscle which are essential for normal muscle contraction and relaxation. Proteins associated with cell proliferation and inflammation were influenced which may account for some of the functional known deficits that occur in diabetic complications of bladder [20]. Even if the investigation of the initiation, development and progression of the bladder dysfunction in a high fat diet (HFD) induced obese animal model has started [21] our understanding of the underlying molecular mechanism is still incomplete.

Thus, we applied a label-free quantitation as an unbiased strategy to identify pathways involved in urinary bladder metabolism in a HFD rat model. Based on the Ingenuity pathway analysis (IPA) we developed a hypothetical model of the pathomechnism of bladder dysfunction. To support our hypothesis we analysed key proteins of the identified pathways in separate validation cohorts by independent analyses of protein abundance, activity and tissue localization. To date specific therapeutic strategies for obesity related bladder dysfunctions are rare. The discovery of cell specific targets may provide novel therapeutic options.

## Methods

### Experimental Animals

All animal procedures were approved by the “Landesdirektion” Leipzig (TVV 32/11) and were carried out in strict accordance with the recommendations of the German “Tierschutzgesetz” (TierSchG). We randomized 30 male four weeks old Sprague-Dawley (MEZ, Medical Experimental Center, University of Leipzig, Leipzig, Germany) rats into two groups: chow diet (CD, energy from fat 11%, carbohydrate 66%, protein 23%; ssniff-Spezialdiaeten GmbH; Soest, Germany) or high fat diet (HFD, energy from fat 45%, carbohydrate 35%, protein 20%) for 11 weeks (diet composition reported in Table S4). Animals were kept on a 12∶12 h light–darkness cycle. Weight gain and food intake were monitored twice a week. Before euthanization, individual urine samples were collected in a metabolic cage over 24 hrs. Metabolic profiles of those animals were recorded on the day of euthanization.

### Histology, Immunostaining and Analysis

Bladder tissue was fixed in 4% formalin over night and subsequently embedded in paraffin. Bladder histology was evaluated in sections stained according to Crossmon [22]. Grade of fibrosis was assessed by van Gieson’s staining [22]. DAB-immunolabelling of eNOS, calpain 2 and HIF1-α was performed using adequate specific primary antibodies (Table S1).

### Cell Culture

Primary cell cultures were set up from small muscle layer fragments of the bladder dome after removing the urothelium with a cotton swab according to Barendrecht *et al*. [23]. Cells were grown in RPMI 1640 medium containing 10% FCS and 1×Penicillin-Streptomycin (Gibco, Life Technologies, NY, USA) in a humidified atmosphere containing 5% CO_2_ at 37°C. At 80 to 90% confluence cells were harvested with UT-buffer containing 8 M urea, 2 M thiourea and sonicated. The homogenates were centrifuged at 14.000 g for 10 min and the supernatant was collected. Protein concentration was determined using a Bradford assay with bovine serum albumin as standard protein (Pierce, Thermo Scientific).

### Tissue Destruction, Proteolytic Digestion and nano LC-MS/MS Analysis

Bladder tissues were snap frozen in liquid nitrogen and powdered using a Mikro Dismembrator (Braun, Melsungen, Germany) at 2600 rpm for 2 min with 150 µL UT. The tissue powder was reconstituted in 1.3–2.7 mL of UT and sonicated on ice three times for 3–5 s each with nine cycles at 80% energy using a Sonoplus (Bandelin, Berlin, Germany). The homogenates were centrifuged at 16,200 g for 1 h at room temperature. The supernatant was collected and the protein concentration was determined using the Bradford assay. The protein lysates of the rat bladders were analysed by shotgun LC-MS/MS as described earlier [24]. Proteolytically cleaved peptides (400 ng) were separated prior to mass spectrometric analyses by reverse phase nano HPLC on a 15 cm Acclaim PepMap100-column (C18, 3 µm, 100 Å) using an EASY-nLC Proxeon system (Thermo Scientific, Waltham, MA) at a constant flow rate of 300 nL/min. Separation was achieved using a non-linear gradient of 86 min with 0.1% acetic acid, 2% acetonitrile in water (solvent A) and 0.1% acetic acid in 100% acetonitrile (solvent B). Separated peptides were monitored using an LTQ Orbitrap Velos MS (Thermo Scientific). Raw data from the LTQ Orbitrap Velos instrument was processed using the Refiner MS 7.5 and Analyst 7.5 module (Genedata, Basel, Switzerland). Generated peak lists were searched against a rat’s FASTA-formatted database containing 7,928 unique entries (rattus_uniprot_swissprot_2012_10.fasta) using an in-house Mascot server 2.3.2 (Matrix Science, London, GB). Database searches were performed with carbamidomethyl (C) as fixed modification and oxidation (M) as variable modification. Enzyme specificity was selected to trypsin with up to two missed cleavages allowed using 10 ppm peptide ion tolerance and 20 mmu MS/MS tolerance. Only ranked one peptide hits and a Mascot ion score >23 were considered as identified. After peak annotation, the data were further processed in Analyst 7.5, where statistical data evaluation was performed.

### Dot Blotting, Western Blotting, ELISA and Activity Assays

To quantify abundance and activity of proteins of interest we used Dot- and Western blotting and performed ELISA and activity assays (Table S1, Table S2) on HFD (n = 10) and CD (n = 10) rats. Tissue was homogenized with Ultra Turrax (VWR International, Darmstadt, Germany), sonicated on ice three times for 3–5 s each with nine cycles at 80% energy using a Sonoplus (Bandelin, Berlin, Germany). The homogenates were centrifuged at 14,000 g for 10 min and protein concentration was determined (Bradford assay).

Protein carbonylation in bladder tissue homogenate was quantified using 2,4-Dinitrophenylhydrazin (DNPH) derivatisation and Dot-blot [25] indirect immunofluorescence detection and quantification with an Odyssey Infrared Imager (Li-Cor Biosciences). Total protein was visualized by SYPRO Ruby blot stain (BioRad, Munich, Germany). 14-3-3η, eNOS and phosphorylated eNOS (Ser 1177) were validated by Western blotting (Table S1). To quantify 14-3-3η, eNOS and phosphorylated eNOS the blots were analysed by densitometry (1-D analysis software package, Scanalytics, Billerica, Massachusetts). For activity assays and ELISAs used refer to Table S2.

### Immunohistochemistry

Paraffin bladder tissue slices were incubated overnight at 4°C with primary antibodies (Table S1). Indirect labelling was performed using DAB after incubation with adequate secondary antibodies conjugated with horseradish peroxidase for 1 hour at room temperature.

### Semi-quantitative Confocal Immunofluorescence of eNOS and HIF1-alpha

Paraffin bladder tissue slices were incubated overnight at 4°C with primary antibodies (Table S1). Indirect immunofluorescence was performed with adequate fluorescence labelled secondary antibodies. Cell nuclei were stained with TOPRO (Molecular Probes, Life Technologies GmbH, Darmstadt, Germany). The tissues were analysed at a Zeiss LSM-5 Pascal confocal laser scanning microscope. Multitrack scanning avoided ‘bleeding through’ of the fluorescence in double-labelling experiments. To ensure comparability of fluorescence signal intensity between the samples, we calibrated the detection system on control stains with no primary antibody.

Confocal images of immunolabeled urothelium and detrusor muscle were analysed with ImageJ [26] using self written macros.

### Statistical Analysis

Data analysis was performed using Prism v5.0 (GraphPad Software, La Jolla, USA). Statistical differences were calculated by ANOVA or independent Student t-test. A P-value ≤0.05 was considered statistically significant. Identified proteins were functionally assigned to gene ontology categories and for canonical pathway analysis using Ingenuity Pathway Analysis software (IPA, Ingenuity Systems [27]). The data are shown as mean ± SD. The dots represent the single values. Significant differences between the dietary groups are indicated by bars (p<0.05).

## Results

### Phenotype Characteristics of Dietary Groups

**Figure 1:**
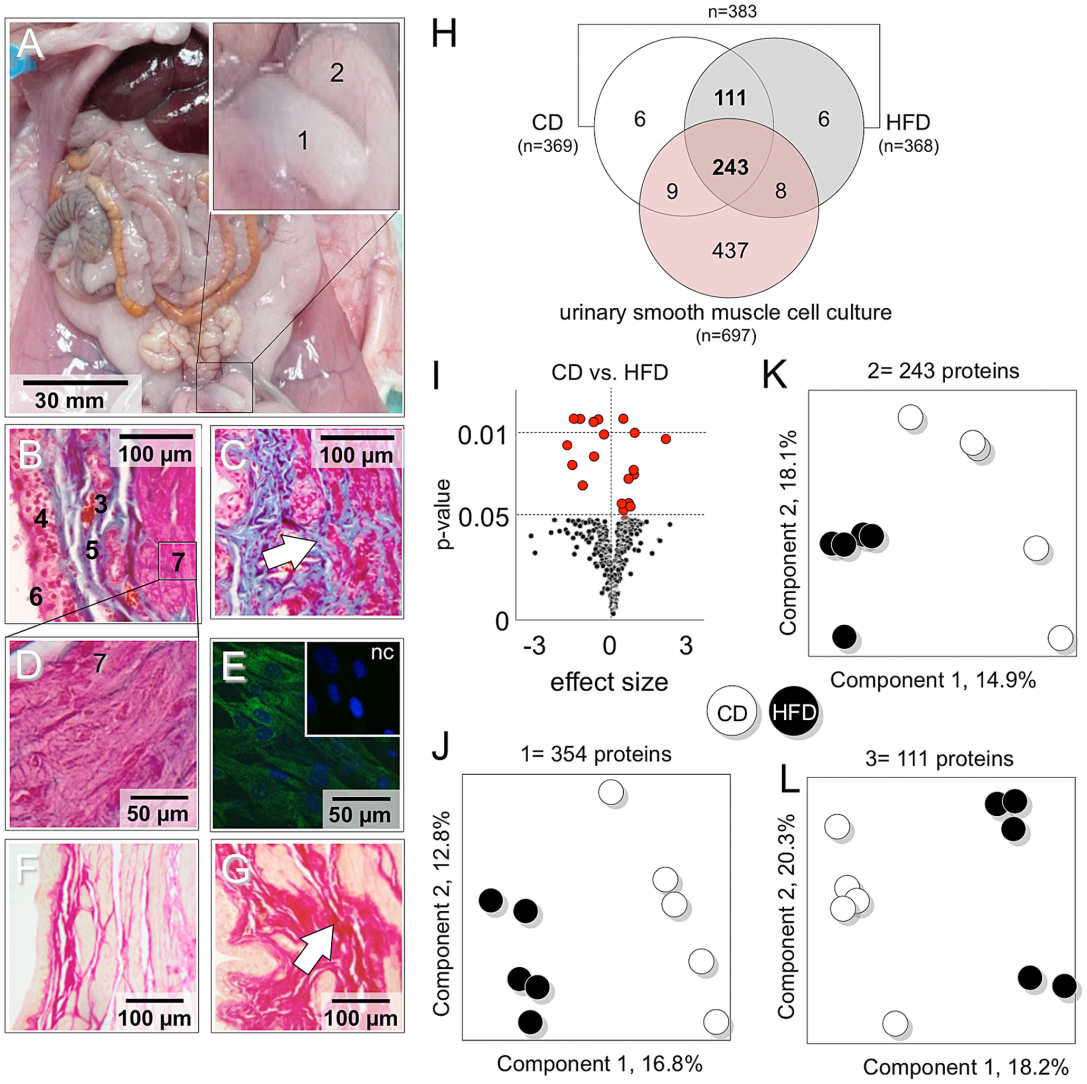
Proteomic analysis of the rat urinary bladder and urinary smooth muscle cell culture. Images show morphology and position of the bladder in the pelvis (A; 1– urinary bladder; 2– prostate). Crossmon staining reveals the complex architecture of bladder wall (B; 3 - blood vessel; 4– the urothelium; 5– suburothelial layer; 6– bladder lumen, 7– detrusor tissue) including detrusor muscle of CD-rats (7; D). In HFD Crosmon staining show accumulation of extracellular matrix (C, arrow). (E) Cultured detrusor myocytes from rat bladder tissue express alpha-smooth muscle cell actin (green). Nuclei are stained with DAPI. Inset represents negative stain control. (F–G) Van Gieson stain revealed collagen infiltration and fibrosis in HFD (G, arrow) compared to CD (F). (G–K) Partial least square (PLS) analysis: Proteome of CD and HFD-bladder and detrusor myocytes culture were studied by LC-MS/MS. (G) Venn diagram display distribution of regulated proteins obtained from the comparison of LC-MS/MS. (H–L) PLS was used to classify diet induced alteration of cellular protein expression profiles. (I) Volcano plot displayed significant altered proteins in HFD (n = 18). The dietary groups show clear separation of their protein profile based on 354 proteins identified in bladder tissue (J) as well based on 243 proteins specific for detrusor muscle cells (K) and based on 111 proteins related to other tissue than detrusor (L).

Complete phenotypic characteristics of both dietary groups exhibited differences in most relevant parameters including fat masses, homeostatic model assessment-insulin-resistance (HOMA-IR), and plasma IL-6 and UA levels (Table 1). HFD feeding resulted in a significant decrease of voided volume (VV) collected over 24 h in metabolic cages, while creatinine clearance (CrC) remained unchanged (Table 1). The daily and weekly water intake did not differ between both groups (Figure S1A). The free fatty acid profile was significantly different in both groups (Table S1) and a significant negative correlation between 24 h voiding volume to plasma palmitate level was evident (Figure S1B). Bladders from HFD animals showed marked structural changes, e.g. fibrosis in detrusor and lamina propria as revealed by van Gieson staining (Figure 1A–G).

**Table 1:** Clinical characteristics of the study population phenotype.

mean (SD)	Discovery set			Validation set		
	CD n = 5	HFD n = 5	p- value*	CD n = 10	HFD n = 10	p-value*
**body weight [g]**	418±60	519±64	<0.03	430±26	533±24	<0.001
**visceral body fat [g]**	8.6±4.1	13±5.6	ns	6±3.4	11.7±3.5	<0.002
**subcutaneous body fat [g]**	5.5±2.3	8.6±4	ns	5.3±4	9.6±3.2	<0.016
**fasting glucose [mmol/L]**	4.5±0.5	5.7±0.7	<0.013	5.5±0.6	6.3±0.9	<0.031
**fasting insulin [ng/ml]**	1.2±0.11	2.4±0.94	<0.021	1.25±0.14	2.08±0.78	<0.004
**HOMA-IR**	1.2±0.2	3±1.4	<0.018	1.5±0.3	2.9±1.4	<0.005
**creatinine clearence [ml/min]**	1.02±0.32	1.16±0.26	ns	0.99±0.19	1.06±0.35	ns
**24 h urin volume [ml]**	9.2±2.8	4.8±0.4	<0.01	10.5±3.5	6.4±1.8	<0.004
**uric acid [µmol/L]**	74±21.1	123.4±14	<0.002	83.8±27.5	119.7±42	<0.036
**interleukine 6 [pg/ml]**	238±19	269±18	<0.01	223±40	269±32	<0.032
**c-reactive protein [µg/ml]**	324±69	418±58	<0.05	378±63	522±30	<0.001
**triglyceride [mmol/L]**	0.83±0.55	1.76±0.59	<0.033	0.69±0.49	1.3±0.75	<0.045
**total-cholesterol [mmol/L]**	2.44±0.82	4.72±1.66	<0.025	2.79±1.03	3.88±0.86	<0.045
**HDL-cholesterol [mmol/L]**	1.24±0.47	1.36±0.39	ns	0.97±0.25	1.29±0.44	ns
**LDL-cholesterol [mmol/L]**	1.42±1.13	2.54±1.29	ns	1.41±0.83	2±0.51	ns

Clinical characteristics of the dietary groups after 15 weeks. Values are means ± SD for discovery set and validation set; CD - chow diet; HFD - high fat diet; HOMA-IR - homeostatic model assessment of insulin resistance. The HOMA-IR was calculated by multiplying fasting plasma glucose (mg/dl) by fasting plasma insulin (2 U/ml) divided by 2,430 according to the method described by Cacho et al. [52]. LDL-cholesterol was calculated by Friedewald’s formula: LDL = TC - HDL - TG/2.2 (mmol/L) [53]. Urinary uric acid was measured by high-performance liquid chromatography. Assays used are listed in supplemental Table S2.

### Urinary Bladder Proteome Analysis

Comparative proteomic profiling of the HFD induced protein changes in rat urinary bladder was performed applying a label free shotgun proteome approach (Figure 1H–L). We identified 383 proteins in the urinary bladder tissue of 5 CD and 5 HFD rats. 354 (111+243) of these proteins were expressed in all animals and were included in quantitative analysis (Figure 1H). 18 proteins were significantly regulated (t-Test, P≤0.05; Volcano plot in Figure 1I) including calpain-2, 14-3-3η, and peroxiredoxin-1 (Table 2). Partial least square (PLS) analysis separated CD and HFD groups (Figure 1J).

**Table 2:** Differentially abundant proteins identified by LC-MS/MS.

Protein	Uniprot	Description	Unique peptides	p-Value	Effect size	Fold changeHFD/CD	Cellular component	Biological process
**Proteins found in rat urinary bladder tissue and SMC culture**								
IMPA1	P97697	Inositol monophosphatase 1	2	0.016	1.573	0.06	1, 2, 3, 4	12, 13, 14
CAN2	Q07009	Calpain-2	1	0.033	0.463	0.32	1, 2, 5, 10	12, 13, 15, 16
1433F	P68511	14-3-3η	1	0.044	0.471	0.32	1	13, 14, 15, 16, 17, 18
OST48	Q641Y0	Dolichyl-diphosphooligo-saccharide–protein glycosyl-transferase 48 kDa subunit	1	0.031	0.629	0.376	1, 5, 6	12, 13
ODPB	P49432	Pyruvate dehydrogenase E1 component subunit beta, mitochondrial	3	0.012	0.646	0.411	1, 3, 4	12, 14
EF2	P05197	Elongation factor 2	8	0.006	0.305	0.571	1	12
TBB2A	P85108	Tubulin β-2A chain	1	0.045	0.279	0.61	1, 11	12, 17
PRDX1	Q63716	Peroxiredoxin-1	4	0.047	0.301	0.743	1, 2, 3, 4, 7, 10	12, 13, 14, 18, 19, 20, 21, 22, 23
PGAM1	P25113	Phosphoglycerate mutase 1	1	0.013	−0.267	2.019	1, 2, 7	12, 14
LUM	P51886	Lumican	9	0.006	−0.451	2.113	10	13, 15
G6PD	P05370	Glucose-6-phosphate 1-dehydrogenase	1	0.007	−0.6	2.469	1, 2, 5, 7, 11	12, 13, 14, 15, 16
GSTP1	P04906	Glutathione S-transferase P	2	0.023	−0.559	2.978	1, 2, 5, 7	12, 13, 14, 15, 16, 19, 20, 21
**Proteins found only in rat urinary bladder tissue**								
RS21	P05765	40S ribosomal protein S21	1	0.029	0.6	0.302	1, 7, 8	12
DHE3	P10860	Glutamate dehydrogenase 1, mitochondrial	7	0.05	0.347	0.508	1, 3, 4, 5	12, 13, 14, 18, 21
THIM	P13437	3-ketoacyl-CoA thiolase, mitochondrial	1	0.036	−0.901	2.565	1, 3, 4, 5	12, 14, 19
SVS2	P22006	Seminal vesicle secretory protein 2	13	0.006	−1.009	5.728	1, 10	13, 16, 24
SPA3K	P05545	Serine protease inhibitor A3K	5	0.006	−1.148	12.798	10	12, 13, 14
GNAO	P59215	Guanine nucleotide-binding protein G(o) subunit α	2	0.027	−1.226	19.62	5	12, 13, 14, 15, 16, 17, 18

Untargeted shotgun proteome approach revealed 18 significant regulated proteins comparing HFD (n = 5) to CD rats (n = 5). 1 cytoplasm; 2 nucleus; 3 mitochondrion; 4 organelle lumen; 5 membrane; 6 endoplasmic reticulum; 7 cytosol; 8 ribosome; 9 chromosome; 10 extracellular; 11 cytoskeleton; 12 metabolic process; 13 response to stimulus; 14 regulation of biological process; 15 development; 16 cell differentiation; 17 cell organization and biogenesis; 18 transport; 19 cell death; 20 cell proliferation; 21 cell communication; 22 defense response; 23 cellular homeostasis; 24 reproduction.

### Proteome Analysis of Cultured Rat Detrusor Cells

In pilot studies we explored the possibility of separate proteomic analysis of urothelium and detrusor smooth muscle from native rat bladder. However, the amount of cells recovered from scraping off the urothelium was too small to be used for valid and reproducible proteomic experiments. Since these two cell types are the major components of the bladder and are responsible for different physiological functions, we established cell cultures of isolated bladder smooth muscle cells of CD rats and performed a liquid chromatography-tandem mass spectrometry (LC-MS/MS) proteome approach. In total we identified 697 proteins of which 243 proteins were also identified in CD and HFD rat urinary bladder tissue (Figure 1H). 243 proteins of the 383 proteins that were found in bladder tissue are prototypic for bladder smooth muscle cells. Alterations in the expression to those proteins may reflect significant functional changes. PLS analysis of these 243 proteins in rat bladder tissue revealed a clear separation of CD and HFD animals (Figure 1K). The remaining 111 proteins monitored in the proteome analysis of bladder tissues (Figure 1H) likely represent other tissue components, such as urothelium and extracellular matrix. Variance in those 111 proteins also separated CD and HFD animals in a PLS analysis (Figure 1L).

### Functional Pathway Analysis

**Figure 2:**
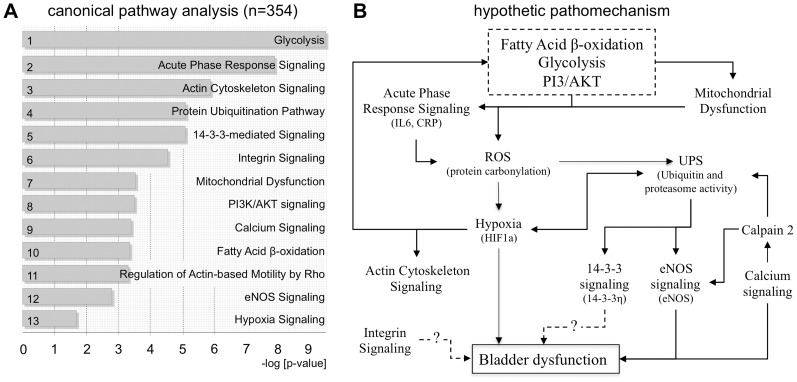
Canonical pathway analysis of identified rat bladder proteins. (A) Based on 354 proteins of rat bladder wall tissue, the canonical pathway analysis revealed dysregulation of pathways regulating metabolic, inflammatory, structural processes as well as cellular signalling in HFD compared to CD. (B) Hypothetic pathomechanism of bladder dysfunction.

Enrichment analysis of proteomic data (n = 354) was performed by Ingenuity Pathway Analysis (IPA). Each enriched category is assigned an adjusted p-value (Fisher’s exact test) and displays the most significant canonical pathways across the entire dataset by searching from several public resources (Figure 2A; Figure S2). Based on the proteomic data and literature reports we designed a hypothetical working model for the potential involvement of the regulated pathways in bladder dysfunction (Figure 2B). It is important to validate such pathways by well-defined signalling key targets, because IPA only depicts hypothetical signalling.

### Targeted Protein Analysis in an Independent Validation Study Population

**Figure 3:**
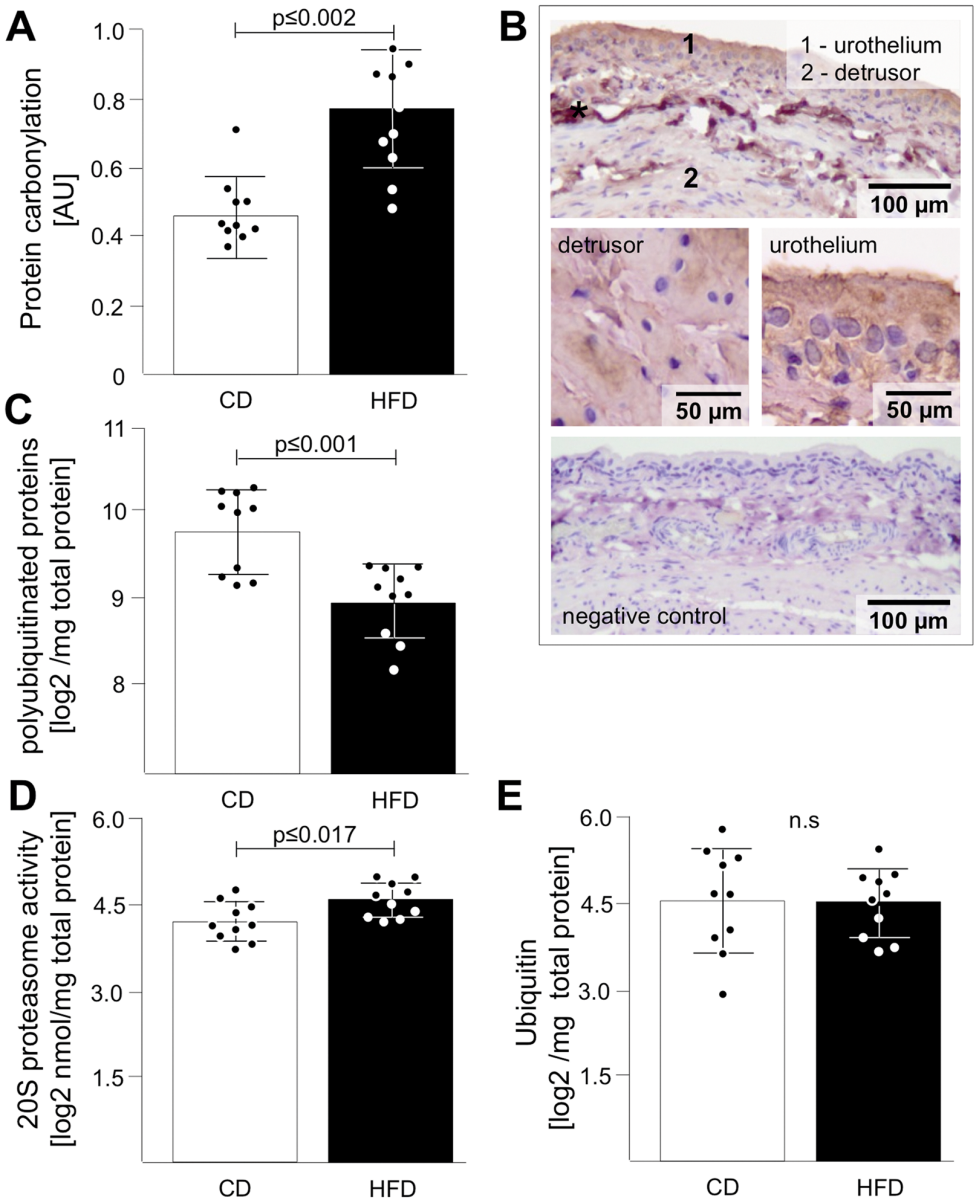
Validation of protein carbonylation and proteasome-ubiquitin pathway. Analysis of protein carbonylation using DNPH-staining on Dot-blot revealed increased abundance of carbonylated proteins in HFD rats (A). (B) DAB staining revealed carbonylated proteins in both, the urothelium (1) and detrusor (2); star indicates cutting artifacts. Imabe below represents negative control without primary antibody). (C) Analysis of protein degradation demonstrated by ubiquitination revealed significant decrease of polyubiquitinated proteins in HFD. Proteasome activity was significantly increased (D) while the ubiquitin amount was not altered (E). Data are shown as mean ± SD. The dots represent the single values. Statistical evaluation by independent Student t-test, p<0.05 was considered as statistical significant.

The proteomic screening revealed an up-regulation of glycolytic and lipid metabolism (Table 1; Table S3) induced by HFD and marked changes in the proteomic pattern of the urinary bladder (Figure 1; Figure S2). Up-regulation of acute phase response signalling was confirmed in serum by increased levels of IL6 and CRP (Table 1) and by histological findings of severe fibrosis (Figure 1F–G). Since IPA only indicates involvement of distinct pathways we validated interesting major pathways in additional animals (10 CD and 10 HFD rats). We expected an induction of ROS via acute phase response and mitochondrial dysfunction (Figure 2B) and in support of this thesis we found higher levels of carbonylated proteins in the HFD compared to CD (Figure 3A) and demonstrated localization of carbonylated proteins in both, urothelium and detrusor without any obvious preference (Figure 3B).

### Ubiquitin Proteasome Pathway (UPS)

One important regulated pathway in HFD rat urinary bladder based on canonical pathway analysis was UPS (Figure 2A; Figure S2). In support we found reduced levels of polyubiquitinated proteins (Figure 3C) and an increase of 20S proteasome activity (Figure 3D) in HFD. However, total free ubiquitin was not affected (Figure 3E).

### Hypoxia

**Figure 4:**
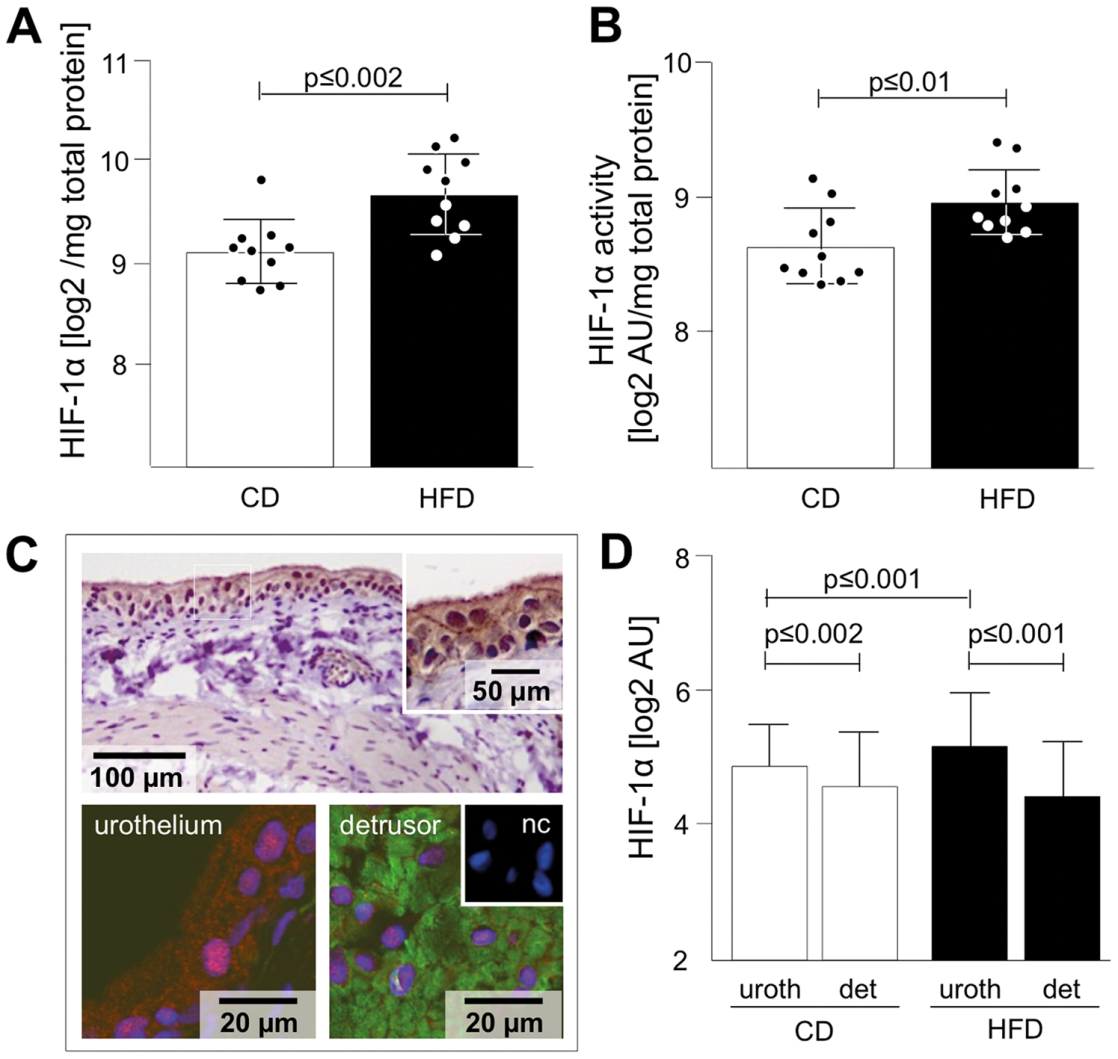
Validation of HIF-1α abundance and activity. HIF-1α, the indicator for Hypoxia, showed increased abundance (A) and activity (B) in HFD rats measured by ELISA. (C) DAB staining revealed HIF-1α in both, the urothelium und detrusor showing strong membrane labelling. HIF-1α abundance (red) was quantified by confocal immunofluorescence of urothelium and detrusor; nuclei are stained with DAPI (blue); detrusor express alpha actin (green); nc – negative control. (D) Analysis of confocal immunofluorescence showed higher HIF-1a expression in the urothelium (uroth) compared to detrusor (det), (n = 105 ROIs each column). Data are shown as mean ± SD. The dots represent the single values. Statistical evaluation by independent Student t-test, p<0.05 was considered as statistical significant.

IPA revealed proteins involved in hypoxia signalling (Figure 2A). The key-protein regulated under hypoxic conditions is HIF-1α and we found higher levels of HIF-1α and increased HIF-1α activity in HFD compared to CD (Figure 4A–B). Immunohistochemistry revealed preferential localization of HIF-1α in the urothelium (Figure 4C). Semiquantitative analysis of confocal immunofluorescence showed higher HIF-1α abundance in urothelium of both, CD and HFD. Interestingly HIF-1α abundance was significantly higher in the urothelium of HFD (Figure 4D).

### Endothelial Nitric Oxide Synthase (eNOS)

**Figure 5:**
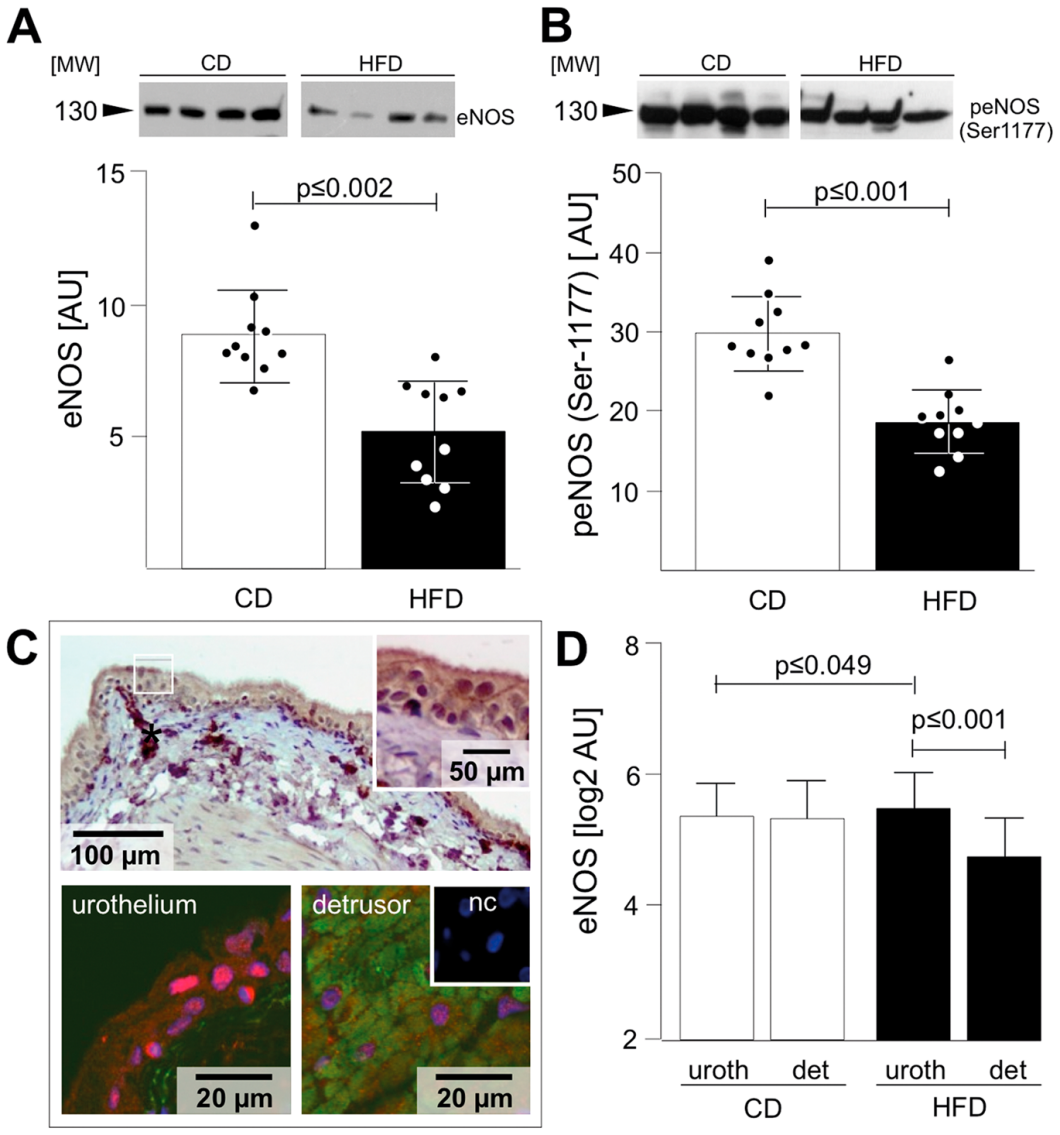
Validation of eNOS abundance and phosphorylation. Western Blot analysis of eNOS showed decreased abundance of eNOS (A) and phospho eNOS 1177 (B) in HFD rats. (C) DAB staining revealed eNOS in both, the urothelium and detrusor. ENOS abundance (red) was quantified by confocal immunofluorescence of urothelium and detrusor (insets are 3× magnification from same image; nuclei are stained with DAPI (blue); detrusor express alpha actin (green); nc – negative control); star indicates cutting artifacts. (D) Analysis of confocal immunofluorescence showed higher eNOS expression in the urothelium (uroth) of HFD rats compared to their detrusor (det) while eNOS expression in CD rats did not differ between the urothelium and detrusor (n = 105 ROIs each column). Data are shown as mean ± SD. The dots represent the single values. Statistical evaluation by independent Student t-test, p<0.05 was considered as statistical significant.

Involvement of the eNOS signalling pathway was confirmed by determining reduced levels of total eNOS (Figure 5A) and phosphorylated (activated) eNOS (Ser-1177; Figure 5B) in HFD vs. CD. The ratio of phosphorylated eNOS/total eNOS was not significantly altered. Confocal immunofluorescence analysis showed equal distribution of eNOS in urothelium and detrusor of CD, whereas eNOS level was increased in urothelium but decreased in detrusor of HFD (Figure 5C–D).

### 14-3-3 Signalling

**Figure 6:**
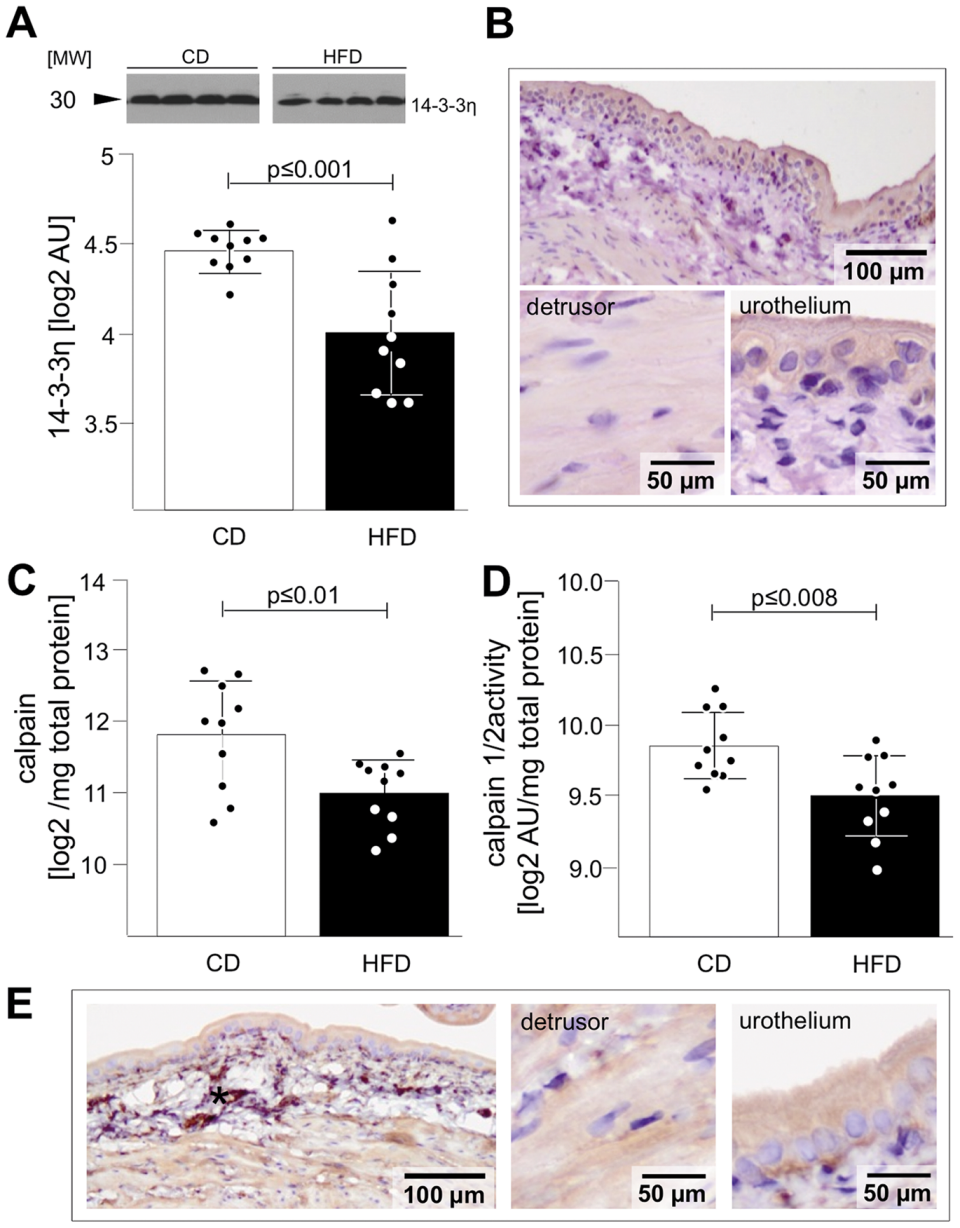
Validation of 14-3-3η and calpain 2. Western Blot analysis of 14-3-3η showed decreased abundance (A) in HFD rats. (B) DAB staining revealed 14-3-3η in both, the urothelium und rBSMC. (C-E) Analysis of calpain 2 showed down-regulation of calpain 2 expression (C) and calpain 1/2 activity (D) in HFD rats. DAB staining revealed eNOS in both, the urothelium und rBSMC (E); star in (E) indicates cutting artifacts. Data are shown as mean ± SD. The dots represent the single values. Statistical evaluation by independent Student t-test, p<0.05 was considered as statistical significant.

The polypotent 14-3-3-mediated signalling pathway was statistically relevantly regulated in IPA comparing CD and HFD (Figure 2A). Additionally, the comparison via Student t-Test of CD and HFD proteome revealed significant down-regulation of 14-3-3η in HFD (Table 2). Consequently, we confirmed these findings by Western blot analysis (Figure 6A). Immunohistochemistry showed equal distribution of 14-3-3η in urothelium and detrusor (Figure 6B).

### Calcium Signalling

IPA revealed differences in calcium signalling in both groups (Figure 2A) and Student`s t-Test showed reduced levels of calcium-dependent, non-lysosomal cysteine protease (calpain-2; Table 2). We confirmed the reduced calpain-1/2 abundance and activity in HFD vs. CD (Figure 6C–D). Immunohistochemistry showed equal staining intensity of Calpain-2 in urothelium and detrusor (Figure 6E).

## Discussion

Several important messages emerge from our present study: i) the proteome pattern of urinary bladder significantly changes under HFD conditions; ii) several major pathways known to be involved in bladder function are regulated; iii) differential expression of several key proteins of those pathways in urothelium and detrusor indicate differential susceptibility of those cell types to changes in lipid metabolism.

It is well known, that obesity is strongly related to bladder dysfunction [12] and Gasbarro et al. recently provided evidence that obesity without disturbed glucose metabolism alters bladder function by reducing urodynamic pressure and volume in rats [4]. However, except for one proteomic study on streptozotocin-induced (STZ) diabetes mellitus rats no data are available about HFD induced changes of urinary bladder metabolism [20].

To gain a deeper insight into the pathophysiology of obesity-related bladder dysfunction we designed a rat HFD obesity model, allowing control of diet and environmental conditions, and analysed changes in the proteome of the whole bladder by shotgun proteomic approach and validated our hypothetical pathophysiological pathway model by measuring the levels of key proteins in separate animal cohorts. Histological evaluation was undertaken to pinpoint those changes to distinct cell types of the rat urinary bladder, i.e. urothelium and detrusor smooth muscle cells. Interestingly, proteome analysis using IPA showed that glycolysis was the most prominent up-regulated pathway in urinary bladder of HFD and disturbance of glucose tolerance was detected in plasma by intraperitoneal glucose tolerance test. In addition we found an increase of free fatty acid (FFA) profile, especially of saturated FFA palmitate, which is known to induce release of the acute phase response related protein (IL-6) in cultured human bladder smooth muscle cells [15]. Furthermore, palmitate can also alter mitochondrial function as we showed recently [14]. Intriguingly, those three pathways: glycolysis, fatty acid β-oxidation, and mitochondrial dysfunction were detected to be altered in HFD by proteome analysis. Up-regulation of those pathways is well-known to induce ROS [28], [29]. ROS are able to modify protein function by carbonylation. We identified significant increase of carbonylated proteins in HFD urinary bladder providing a link to cytoskeletal signalling, which was one major significantly altered pathway in IPA [30].

### Acute Phase Response Signalling

Acute phase response signalling is the most prominent regulated pathway by IPA in HFD urinary bladder. Serum parameters of acute phase reaction such as IL-6 and CRP significantly increased. We found up-regulation of glycolysis and lipid metabolism and up-regulation of acute phase response signalling including increased levels of IL6, demonstrating that those mechanisms are relevant in HFD induced bladder pathophysiology. Recent studies showed that acute phase mediators, such as interleukins were expressed in urinary bladder tissue and may cause bladder wall irritation such as fibrosis [31]. In the present study, histological staining of bladder wall tissue confirmed morphological alterations such as fibrosis and increased collagen in HFD rats. Thus, structural changes based on inflammation and lower efficiency of anti-inflammatory processes might contribute to urgency, frequency and increased resting volume, typically seen in obesity related urinary bladder dysfunction [4]. Local inflammation has been proposed to be closely related to hypoxia in obese phenotype [32].

### Hypoxia

IPA revealed down-regulation of proteins such as HS90A/B and ENPL, involved in hypoxia signalling in HFD rats. Especially HSPs are known to be linked to hypoxia signalling, by HIF-1α-dependent increase of HSPs as a functional impact to maximize production of protective HSPs. In addition it has been shown that insulin resistance in adipocytes is dependent on HIF-1α transcription factor expression [33]. HIF-1α is under control of two classes of oxygen-dependent enzymes, which by hydroxylation induce ubiquitination of HIF-1α and lead to HIF-1α degradation. The reactive oxygen species H_2_O_2_ inhibits activity of those regulatory enzymes and thus triggers increase of HIF-1α under hypoxic conditions [34]. We found increased abundance and activity of HIF-1α in HFD bladder tissue and differential expression and regulation of HIF-1α in urothelium and detrusor. Our findings indicate that urothelium is especially vulnerable to hypoxia induced by HFD. This novel finding is especially intriguing for a hypothetical pathomechanism leading to bladder dysfunction. Our notion is strongly supported by a recent study demonstrating increased expression of HIF-1a, HIF-2a and VEGF expression upon stretching in bladder urothelial cells cultured from patients with idiopathic OAB [35]. On the other hand, recent studies showed that hypoxia (followed by re-oxygenation) resulted in time-dependent progressive reduction in contractile responses of bladder strips [36]. This is in line with our findings indicating both down-regulation of actin cytoskeleton signalling and actin-based motility by rho as regulated pathways. Hypoxia as a key signalling promotes pathomechanism of bladder dysfunction and our results showed a relation to HFD induced obesity as a possible mechanism [37].

### Ubiquitin-Proteasome System (UPS)

Hypoxia is able to induce ubiquitin-proteasome system via ROS and otherwise it was shown that the UPS pathway mediates the oxygen-dependent proteolysis of HIF-1α, the transcription factor important in adaptation to hypoxia [38]. To our best knowledge, up to date no study focused on regulation of UPS in HFD related urinary bladder dysfunction. We identified significantly reduced levels of proteins related to UPS pathway. There is growing evidence that ubiquitin-proteasome mediated protein degradation plays a critical role in the regulation of a wide range of proteins and therefore regulate metabolism of cells [38]. Our findings indicate both down-regulation of enzymes responsible for ubiquitination such as ubiquitin-like modifier activating enzyme 1 (UBA1) and down-regulation of proteins involved in proteasome complex such as proteasome activator subunit 1 (PSME1; Figure S2). The up-regulation of UPS pathway can be explained by the increase of ROS mediated carbonylation of proteins and hypoxia [34], [39]. Despite the fact that HIF-1α as a marker for hypoxia was especially enhanced in urothelium of HFD, up-regulation of carbonylated proteins equally affected both, urothelium and detrusor. Consequently, induction of proteasomal degradation by increase of protein carbonylation also enhances degradation of other proteins. We found decrease in several key proteins describing important pathways for metabolic bladder function, such as calpain, eNOS, and 14-3-3.

### Calpain Signalling

Calpains are calcium dependent proteases, involved in vital cell functions such as cell motility and tissue renewal [40]. Calpain can lyse specific membrane proteins and has been shown to mediate ischemia/reperfusion and partial bladder outlet obstruction induced bladder dysfunction [41]. Additionally, calpain is known to increase proteasome-dependent proteolysis and inhibiting protein synthesis [42]. We observed significant reduction of calpain (CAN2) abundance and activity in HFD compared to CD with no difference in tissue distribution. This may indicate a counter regulation mechanism to increased UPS [42].

Furthermore, calpain activation was also shown to reduce eNOS activity [43]. Down-regulation of calpain might reflect general regulation of calcium signalling. Pathological calcium signalling may directly affect contractile properties of detrusor smooth muscle cells. Detrusor contraction is counteracted by nitric oxide release from urothelium mediating relaxation of the bladder during storage phase [44]. Typically, storage phase is disturbed in obesity phenotype and has been shown in rat obesity model previously [4].

### eNOS Signalling

Nitric oxide (NO) is an important modulator of bladder tone and contributes to local immune defence [45]. Decreased eNOS activity is linked to hypoxia and NO signalling plays an essential role in the pathomechanism of bladder dysfunction [46]. Our data displayed a down-regulation of the eNOS signalling pathway and we further verified decreased phosphorylated eNOS in HFD rats by western blotting.

Recent findings indicate that changes in NO production and impaired NO control are early events in bladder dysfunction of diabetes phenotype [45] and our results support a causal relationship of impaired NO signalling in the detrusor and metabolic bladder dysfunction in HFD rats. We found equal distribution of eNOS in urothelium and detrusor in CD rat bladders. However, in HFD eNOS expression was significantly increased in urothelium but remarkably decreased in detrusor. This confirms the hypothesis, that impaired NO signalling of both urothelium and detrusor play a critical role in the development of obesity related bladder dysfunction. Interestingly, a recent study demonstrates, that urothelial production of NO and relaxation of the detrusor muscle is impaired in insulin resistant diabetic mice [47]. The authors further provide evidence for endoplasmatic stress as a mechanism for the development of non-voluntary detrusor contractions often seen in diabetic OAB.

### 14-3-3 Pathway

Differential regulation was found for various eNOS pathway related proteins including 14-3-3η [48]. 14-3-3 signalling is a pleiotropic pathway with major involvement in insulin signalling and pancreatic β-cells survival [49]. Up to date this pathway has not been focused in relation to bladder dysfunction, despite it is a central pathway for cell survival, glucose metabolism, tissue development and remodelling [50], [51]. Shotgun proteomics identified both 14-3-3 signalling and especially down-regulation of 14-3-3η in HFD. Both, urothelium and detrusor seemed to be involved, since no difference in 14-3-3η distribution was observed in immunohistochemistry. Down-regulation of 14-3-3 pathway may account for the complex metabolic alterations and structural changes seen in HFD urinary bladder. Further investigations are necessary to explore the relevance of the 14-3-3 pathway for bladder physiology and pathophysiology.

### Conclusions

Increased cellular metabolism leads to increased acute phase reaction, hypoxia and cellular stress. Activation of those pathways might promote increased activity of UPS and secondarily decreases abundance and activity of 14-3-3η, eNOS and CAN2. The context of massive alteration of intracellular signalling results in bladder structural alterations and lower voided urine volume. Further studies on human tissue and cells are required to demonstrate the significance of diet for urinary bladder function. Focused analysis of the identified pathways will provide a valuable approach to reach this goal.

## Supporting Information

Figure S1 **Alterations in water household.** Comparison of CD and HFD rats display no changes in weekly and daily water intake (A). Plasma palmitate level was inverse correlated to 24 urine amount (B).
(TIF)

Figure S2 **Regulation of proteins related to signalling pathways.** Comparison of rat bladder wall proteome revealed plenty of up-regulated proteins in HFD (green) and in CD (red). 1– Glycolysis; 2 - Acute Phase Response Signalling; 3 - Actin Cytoskeleton Signalling; 4 - Protein Ubiquitination Pathway; 5 - 14-3-3-mediated Signalling; 6 - Integrin Signalling; 7 - Mitochondrial Dysfunction; 8 - PI3K/AKT signalling; 9 - Calcium Signalling; 10 - Fatty Acid β-oxidation; 11 - Regulation of Actin-based Motility by Rho; 12 - eNOS Signalling; 13 - Hypoxia Signalling.
(TIF)

Table S1 **Antibodies used for Dot blot (DB), Western blotting (WB), immunohistochemistry (IHC) and indirect immunofluorescence (IF).**
(DOC)

Table S2 **ELISAs and Assays used for validation of target proteins.**
(DOC)

Table S3 **High-fat diet caused massive alteration of free fatty acid profile.** Plasma free fatty acids level of rats undergone chow diet (CD) and high-fat diet (HFD). Values are means ± SD for discovery set and validation set.
(DOC)

Table S4 **Diet composition according to the data sheet information provided by ssniff Spezialdiäten GmbH, Soest, Germany.** CD = chow diet; HFD = high fat diet.
(DOC)

## References

[1] Hammarsten J, Peeker R (2011) Urological aspects of the metabolic syndrome. Nat Rev Urol 8: 483–494. 21811224 10.1038/nrurol.2011.112

[2] Daneshgari F, Moore C (2006) Diabetic uropathy. Semin Nephrol 26: 182–185. 16530610 10.1016/j.semnephrol.2005.09.009

[3] Aizawa N, Homma Y, Igawa Y (2012) Characteristics of lower urinary tract dysfunction and bladder afferent nerve properties in type 2 diabetic Goto-Kakizaki rats. J Urol DOI: 10.1016/j.juro.2012.10.060 10.1016/j.juro.2012.10.060 23103236

[4] Gasbarro G, Lin DL, Vurbic D, Quisno A, Kinley B, et al (2010) Voiding function in obese and type 2 diabetic female rats. Am J Physiol Renal Physiol 298: F72–7. 19889955 10.1152/ajprenal.00309.2009

[5] Wang XM, Zhang MX, Zhao L, Han B, Xu P, et al (2010) The Starling mechanism of the urinary bladder contractile function and the influence of hyperglycemia on diabetic rats. J Diabetes Complications 24: 121–128. 19674919 10.1016/j.jdiacomp.2009.06.002

[6] Lin TL, Chen GD, Chen YC, Huang CN, Ng SC (2012) Aging and recurrent urinary tract infections are associated with bladder dysfunction in type 2 diabetes. Taiwan J Obstet Gynecol 51: 381–386. 23040921 10.1016/j.tjog.2012.07.011

[7] Gomez CS, Kanagarajah P, Gousse AE (2011) Bladder dysfunction in patients with diabetes. Curr Urol Rep 12: 419–426. 21894526 10.1007/s11934-011-0214-0

[8] Ho MH, Yip S, Bhatia NN (2007) Lower urinary tract dysfunctions in women with diabetes mellitus. Curr Opin Obstet Gynecol 19: 469–473. 17885464 10.1097/GCO.0b013e3282efe3b5

[9] Lee WC, Wu HP, Tai TY, Yu HJ, Chiang PH (2009) Investigation of urodynamic characteristics and bladder sensory function in the early stages of diabetic bladder dysfunction in women with type 2 diabetes. J Urol 181: 198–203. 19013605 10.1016/j.juro.2008.09.021

[10] McGrother CW, Donaldson MM, Hayward T, Matthews R, Dallosso HM, et al (2006) Urinary storage symptoms and comorbidities: a prospective population cohort study in middle-aged and older women. Age Ageing 35: 16–24. 16234314 10.1093/ageing/afi205

[11] Kaplan SA, Te AE, Blaivas JG (1995) Urodynamic findings in patients with diabetic cystopathy. J Urol 153: 342–344. 7815578 10.1097/00005392-199502000-00013

[12] Daneshgari F, Liu G, Birder L, Hanna-Mitchell AT, Chacko S (2009) Diabetic bladder dysfunction: current translational knowledge. J Urol 182: S18–26. 19846137 10.1016/j.juro.2009.08.070 PMC4684267

[13] Qatanani M, Lazar MA (2007) Mechanisms of obesity-associated insulin resistance: many choices on the menu. Genes Dev 21: 1443–1455. 17575046 10.1101/gad.1550907

[14] Oberbach A, Schlichting N, Heinrich M, Till H, Stolzenburg J-U, et al (2012) Free Fatty Acid Palmitate Impairs the Vitality and Function of Cultured Human Bladder Smooth Muscle Cells. PLoS ONE PLoS ONE 7: e41026. 22808290 10.1371/journal.pone.0041026 PMC3396599

[15] Oberbach A, Schlichting N, Bluher M, Kovacs P, Till H, et al (2010) Palmitate induced IL-6 and MCP-1 expression in human bladder smooth muscle cells provides a link between diabetes and urinary tract infections. PLoS ONE 5: e10882. 20526368 10.1371/journal.pone.0010882 PMC2878332

[16] Kanda H, Tateya S, Tamori Y, Kotani K, Hiasa K, et al (2006) MCP-1 contributes to macrophage infiltration into adipose tissue, insulin resistance, and hepatic steatosis in obesity. J Clin Invest 116: 1494–1505. 16691291 10.1172/JCI26498 PMC1459069

[17] Lee WC, Chien CT, Yu HJ, Lee SW (2008) Bladder dysfunction in rats with metabolic syndrome induced by long-term fructose feeding. J Urol 179: 2470–2476. 18433789 10.1016/j.juro.2008.01.086

[18] Lee WC, Chuang YC, Chiang PH, Chien CT, Yu HJ, et al (2011) Pathophysiological studies of overactive bladder and bladder motor dysfunction in a rat model of metabolic syndrome. J Urol 186: 318–325. 21600594 10.1016/j.juro.2011.03.037

[19] Rahman NU, Phonsombat S, Bochinski D, Carrion RE, Nunes L, et al (2007) An animal model to study lower urinary tract symptoms and erectile dysfunction: the hyperlipidaemic rat. BJU Int 100: 658–663. 17590178 10.1111/j.1464-410X.2007.07069.x

[20] Yohannes E, Chang J, Christ GJ, Davies KP, Chance MR (2008) Proteomics analysis identifies molecular targets related to diabetes mellitus-associated bladder dysfunction. Mol Cell Proteomics 7: 1270–1285. 18337374 10.1074/mcp.M700563-MCP200 PMC2493381

[21] Buettner R, Parhofer KG, Woenckhaus M, Wrede CE, Kunz-Schughart LA, et al (2006) Defining high-fat-diet rat models: metabolic and molecular effects of different fat types. J Mol Endocrinol 36: 485–501. 16720718 10.1677/jme.1.01909

[22] Romeis B (1989) Mikroskopische Technik. München WienBaltimore: Urban u. Schwarzenberg. p. 1–697.

[23] Barendrecht MM, Mulders AC, van der Poel H, van den Hoff MJ, Schmidt M, et al (2007) Role of transforming growth factor beta in rat bladder smooth muscle cell proliferation. J Pharmacol Exp Ther 322: 117–122. 17435108 10.1124/jpet.106.119115

[24] Hammer E, Goritzka M, Ameling S, Darm K, Steil L, et al (2011) Characterization of the human myocardial proteome in inflammatory dilated cardiomyopathy by label-free quantitative shotgun proteomics of heart biopsies. J Proteome Res 10: 2161–2171. 21417265 10.1021/pr1008042

[25] Robinson CE, Keshavarzian A, Pasco DS, Frommel TO, Winship DH, et al (1999) Determination of protein carbonyl groups by immunoblotting. Anal Biochem 266: 48–57. 9887212 10.1006/abio.1998.2932

[26] Rasband WS ImageJ. U S National Institutes of Health, Bethesda, Maryland, USA. Available: http://rsb.info.nih.gov/ij/, 1997–2006. Accessed: 2013 May 29.

[27] (2012) Ingenuity Pathway Analysis (IPA). Available: http://www.ingenuity.com. Accessed: 2013 May 29.

[28] Johnson RJ, Perez-Pozo SE, Sautin YY, Manitius J, Sanchez-Lozada LG, et al (2009) Hypothesis: could excessive fructose intake and uric acid cause type 2 diabetes? Endocr Rev 30: 96–116. 19151107 10.1210/er.2008-0033 PMC2647706

[29] Aragon JJ, Lowenstein JM (1980) The purine-nucleotide cycle. Comparison of the levels of citric acid cycle intermediates with the operation of the purine nucleotide cycle in rat skeletal muscle during exercise and recovery from exercise. Eur J Biochem 110: 371–377. 7439166 10.1111/j.1432-1033.1980.tb04877.x

[30] San Martin A, Griendling KK (2010) Redox control of vascular smooth muscle migration. Antioxid Redox Signal 12: 625–640. 19737088 10.1089/ars.2009.2852 PMC2829046

[31] Tyagi P, Barclay D, Zamora R, Yoshimura N, Peters K, et al (2010) Urine cytokines suggest an inflammatory response in the overactive bladder: a pilot study. Int Urol Nephrol 42: 629–635. 19784793 10.1007/s11255-009-9647-5

[32] Trayhurn P, Wang B, Wood IS (2008) Hypoxia and the endocrine and signalling role of white adipose tissue. Arch Physiol Biochem 114: 267–276. 18946787 10.1080/13813450802306602

[33] Regazzetti C, Peraldi P, Gremeaux T, Najem-Lendom R, Ben-Sahra I, et al (2009) Hypoxia decreases insulin signaling pathways in adipocytes. Diabetes 58: 95–103. 18984735 10.2337/db08-0457 PMC2606898

[34] Hagen T (2012) Oxygen versus Reactive Oxygen in the Regulation of HIF-1alpha: The Balance Tips. Biochem Res Int 2012: 436981. 23091723 10.1155/2012/436981 PMC3474226

[35] Christiaansen CE, Sun Y, Hsu YC, Chai TC (2011) Alterations in expression of HIF-1alpha, HIF-2alpha, and VEGF by idiopathic overactive bladder urothelial cells during stretch suggest role for hypoxia. Urology 77: 1266.e7–1266.11. 10.1016/j.urology.2010.12.041 PMC3087847 21397301

[36] Whitbeck C, Barreto M, Horan P, Levin SS, Levin RM (1999) Rabbit versus rat urinary bladder: effects of in vitro hypoxia. Pharmacology 59: 156–164. 10450071 10.1159/000028316

[37] Wein AJ (2005) Hypoxia inhibits human bladder smooth muscle cell proliferation: a potential mechanism of bladder dysfunction. J Urol 173: 910. 15711320

[38] Huang LE, Gu J, Schau M, Bunn HF (1998) Regulation of hypoxia-inducible factor 1alpha is mediated by an O2-dependent degradation domain via the ubiquitin-proteasome pathway. Proc Natl Acad Sci U S A 95: 7987–7992. 9653127 10.1073/pnas.95.14.7987 PMC20916

[39] Delobel J, Prudent M, Rubin O, Crettaz D, Tissot JD et al.. (2012) Subcellular fractionation of stored red blood cells reveals a compartment-based protein carbonylation evolution. J Proteomics 76 Spec No.: 181–193. 10.1016/j.jprot.2012.05.004 22580360

[40] Lebart MC, Benyamin Y (2006) Calpain involvement in the remodeling of cytoskeletal anchorage complexes. FEBS J 273: 3415–3426. 16884487 10.1111/j.1742-4658.2006.05350.x

[41] Zhao Y, Levin RM, Levin SS, Wein AJ (1997) Correlation of ischema/peperfusion or partial outlet obstruction-induced spectrin proteolysis by calpain with contractile dysfunction in rabbit bladder. Urology 49: 293–300. 9037302 10.1016/S0090-4295(96)00452-9

[42] Smith IJ, Dodd SL (2007) Calpain activation causes a proteasome-dependent increase in protein degradation and inhibits the Akt signalling pathway in rat diaphragm muscle. Exp Physiol 92: 561–573. 17272355 10.1113/expphysiol.2006.035790

[43] Su Y, Block ER (2000) Role of calpain in hypoxic inhibition of nitric oxide synthase activity in pulmonary endothelial cells. Am J Physiol Lung Cell Mol Physiol 278: L1204–12. 10835326 10.1152/ajplung.2000.278.6.L1204

[44] Burnett AL (1995) Nitric oxide control of lower genitourinary tract functions:A review. Urology 45 No (6) 1071–1083. 10.1016/s0090-4295(99)80136-8 7771014

[45] Poladia DP, Bauer JA (2003) Early cell-specific changes in nitric oxide synthases, reactive nitrogen species formation, and ubiquitinylation during diabetes-related bladder remodeling. Diabetes Metab Res Rev 19: 313–319. 12879409 10.1002/dmrr.385

[46] Birder LA, de Groat WC (2007) Mechanisms of disease: involvement of the urothelium in bladder dysfunction. Nat Clin Pract Urol 4: 46–54. 17211425 10.1038/ncpuro0672 PMC3119256

[47] Leiria LO, Sollon C, Bau FR, Monica FZ, D Ancona CL et al.. (2013) Insulin relaxes human and mice bladder via PI3K/AKT/eNOS pathway activation in mucosal cells: UPR-dependent insulin resistance as a cause of obesity-associated overactive bladder. J Physiol [Epub ahead of print]. 10.1113/jphysiol.2013.251843 PMC3650693 23478138

[48] Jaskille A, Alam HB, Rhee P, Hanes W, Kirkpatrick JR et al.. (2004) D-lactate increases pulmonary apoptosis by restricting phosphorylation of bad and eNOS in a rat model of hemorrhagic shock. J Trauma 57: 262–69; discussion 269–70. 10.1097/01.ta.0000133841.95455.73 15345971

[49] Kleppe R, Martinez A, Doskeland SO, Haavik J (2011) The 14-3-3 proteins in regulation of cellular metabolism. Semin Cell Dev Biol 22: 713–719. 21888985 10.1016/j.semcdb.2011.08.008

[50] Yoon BC, Zivraj KH, Strochlic L, Holt CE (2012) 14-3-3 proteins regulate retinal axon growth by modulating ADF/cofilin activity. Dev Neurobiol 72: 600–614. 21780304 10.1002/dneu.20955 PMC3682208

[51] Thandavarayan RA, Giridharan VV, Sari FR, Arumugam S, Veeraveedu PT, et al (2011) Depletion of 14-3-3 protein exacerbates cardiac oxidative stress, inflammation and remodeling process via modulation of MAPK/NF-kB signaling pathways after streptozotocin-induced diabetes mellitus. Cell Physiol Biochem 28: 911–922. 22178943 10.1159/000335805

[52] Cacho J, Sevillano J, de Castro J, Herrera E, Ramos MP (2008) Validation of simple indexes to assess insulin sensitivity during pregnancy in Wistar and Sprague-Dawley rats. Am J Physiol Endocrinol Metab 295: E1269–76. 18796548 10.1152/ajpendo.90207.2008

[53] Friedewald WT, Levy RI, Fredrickson DS (1972) Estimation of the concentration of low-density lipoprotein cholesterol in plasma, without use of the preparative ultracentrifuge. Clin Chem 18: 499–502. 4337382

