# Supplementary figures and images for: Molecular Fingerprint of High Fat Diet Induced Urinary Bladder Metabolic Dysfunction in a Rat Model
Source: PLoS One. 2013 Jun 24;8(6):e66636. doi: 10.1371/journal.pone.0066636 (PMC3691244; doi:10.1371/journal.pone.0066636)

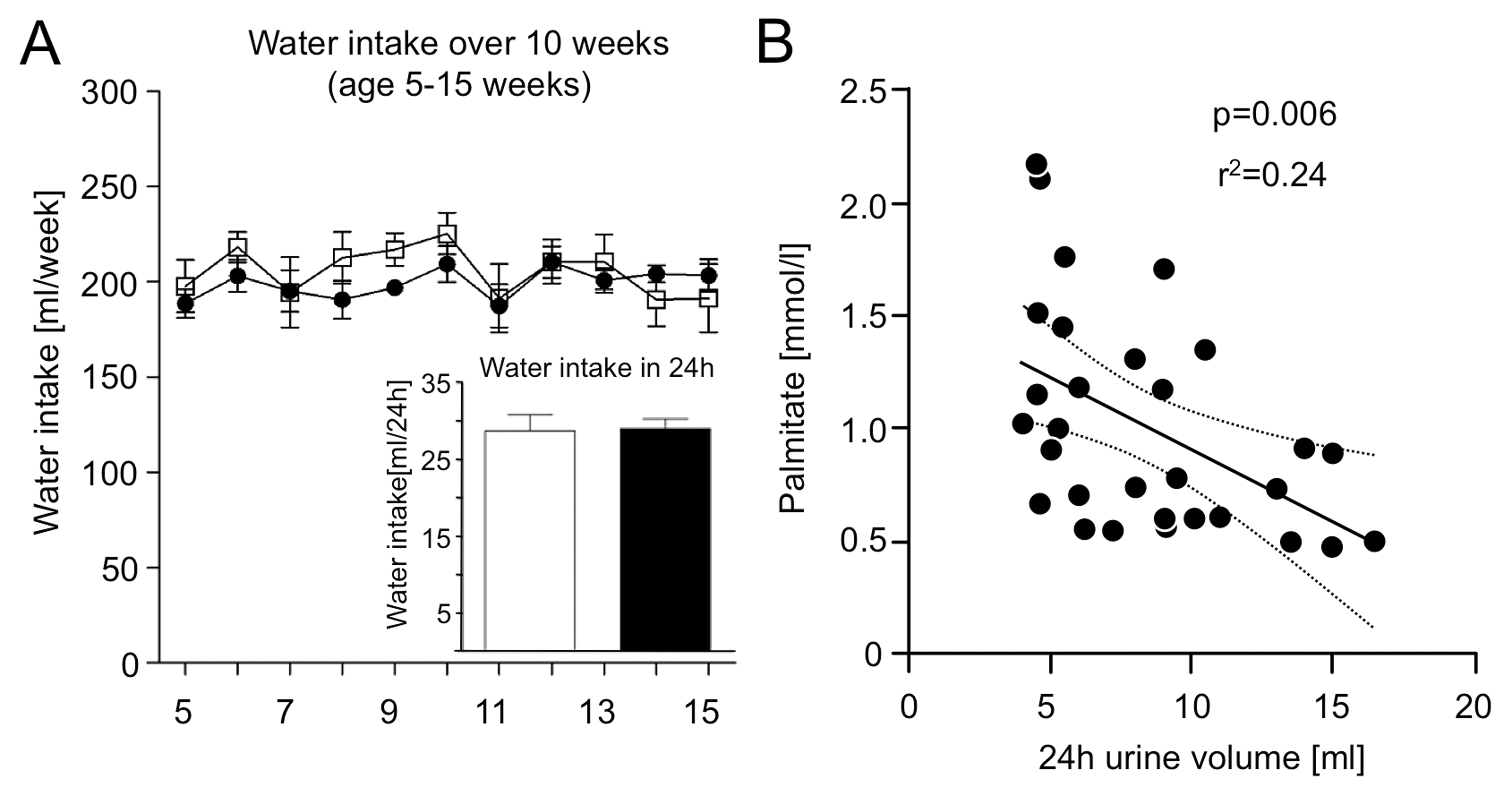

Supplement: Figure S1 — Alterations in water household. Comparison of CD and HFD rats display no changes in weekly and daily water intake (A). Plasma palmitate level was inverse correlated to 24 urine amount (B). (TIF) [file pone.0066636.s001.tif]

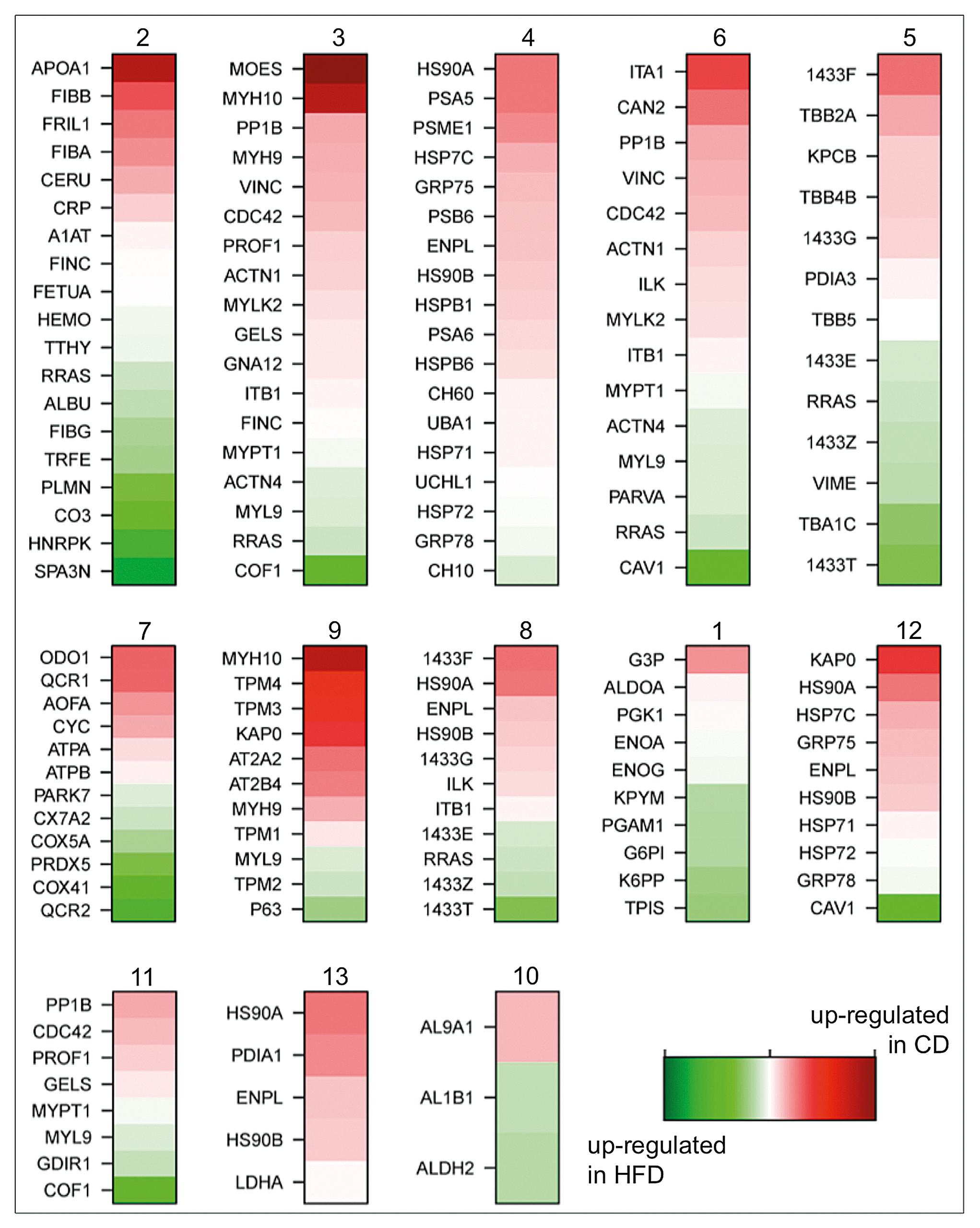

Supplement: Figure S2 — Regulation of proteins related to signalling pathways. Comparison of rat bladder wall proteome revealed plenty of up-regulated proteins in HFD (green) and in CD (red). 1– Glycolysis; 2 - Acute Phase Response Signalling; 3 - Actin Cytoskeleton Signalling; 4 - Protein Ubiquitination Pathway; 5 - 14-3-3-mediated Signalling; 6 - Integrin Signalling; 7 - Mitochondrial Dysfunction; 8 - PI3K/AKT signalling; 9 - Calcium Signalling; 10 - Fatty Acid β-oxidation; 11 - Regulation of Actin-based Motility by Rho; 12 - eNOS Signalling; 13 - Hypoxia Signalling. (TIF) [file pone.0066636.s002.tif]
