# Supplementary material for: Molecular Fingerprint of High Fat Diet Induced Urinary Bladder Metabolic Dysfunction in a Rat Model
Source: PLoS One. 2013 Jun 24;8(6):e66636. doi: 10.1371/journal.pone.0066636 (PMC3691244; doi:10.1371/journal.pone.0066636)
Supplement: Table S1 — Antibodies used for Dot blot (DB), Western blotting (WB), immunohistochemistry (IHC) and indirect immunofluorescence (IF). (DOC) [file pone.0066636.s003.doc]

**Table S1:** Antibodies used for Dot blot (DB), Western blotting (WB), immunohistochemistry (IHC) and indirect immunofluorescence (IF)

| **Primary antibodies** | **host** | **source** | **order-no** | **dilution** |
| --- | --- | --- | --- | --- |
| DNP | mouse | [1] | AM00713PU-N | 1:500 [a] |
| 14-3-3η | goat | [2] | sc-17287 | 1:500 [a]  1:100 [b] |
| HIF-1α | rabbit | [2] | sc-10790 | 1:200 [a-c] |
| NOS3 | rabbit | [2] | sc-654 | 1:200 [a]  1:100 [b-c] |
| eNOS (ps1177) | mouse | [3] | 612392 | 1:2000 [a] |
| alpha-smooth muscle cell actin | mouse, IgG2a | [4] | A2547 | 1:2000 [c] |
| calpain 2 | mouse | [2] | sc-373966 | 1:100 [b] |
|  | | | | |
| **Secondary antibodies / substrates** | | **source** | **order-no** | **dilution** |
| Alexa Fluor® 488 goat anti-mouse IgG2a | | [5] | A-21131 | 1:500 |
| Alexa Fluor® 555 goat anti-rabbit | | [5] | A-21428 | 1:500 |
| IRDye 680 goat anti-mouse IgG1 | | [6] | 926-68021 | 1:5000 |
| SuperSignal West Pico Chemiluminescent Substrate | | [7] | 34077 | n.a. |
| biotinylated goat anti-mouse | | [8] | BA-9200 | 1:500 |
| biotinylated goat anti-rabbit | | [8] | BA-1000 | 1:500 |
| peroxidase coupled donkey anti-goat | | [4] | A5420 | 1:400 |

[1] Acris Antibodies, Herford, Germany

[2] Santa Cruz Biotechnology, Heidelberg, Germany

[3] BD, Heidelberg, Germany

[4] Sigma-Aldrich Chemie GmbH, Steinheim, Germany

[5] Invitrogen, Life Technologies GmbH, Darmstadt, Germany

[6] Li-Cor Biosciences, Bad Homburg, Germany

[7] Thermo Fisher Scientific, Rockford, IL, USA

[8] Vector Laboratories, Burlingame, CA, USA

[a] WB / DB

[b] IHC

[c] IF
