# Supplementary material for: Molecular Fingerprint of High Fat Diet Induced Urinary Bladder Metabolic Dysfunction in a Rat Model
Source: PLoS One. 2013 Jun 24;8(6):e66636. doi: 10.1371/journal.pone.0066636 (PMC3691244; doi:10.1371/journal.pone.0066636)
Supplement: Table S2 — ELISAs and Assays used for validation of target proteins. (DOC) [file pone.0066636.s004.doc]

**Table S2:** ELISAs and Assays used for validation of target proteins

| **ELISA / Assay** | **source** | **order-no** |
| --- | --- | --- |
| Glucose Assay Kit II | [1] | K686-100 |
| Rat Insulin ELISA Kit | [2] | 80-INSRT-E01 |
| Serum Triglyceride Quantification Kit | [3] | STA-397 |
| Total Cholesterol Assay Kit | [3] | STA-390 |
| HDL Cholesterol Assay Kit | [3] | STA-394 |
| Free Fatty Acid Quantification Kit | [1] | K612-100 |
| Creatinine (serum) Assay Kit | [4] | 700460 |
| Creatinine (urinary) Assay Kit | [4] | 500701 |
| Rat IL-6 Quantikine ELISA Kit | [5] | R6000B |
| AssayMax Rat C-reactive protein (CRP) ELISA Kit | [6] | ERC1021-1 |
| Rat Hypoxia Inducible Factor 1а (HIF1а) | [7] | E02H0061 |
| HIF-1а Transcription Factor Assay Kit | [4] | 10006910 |
| Rat Calpain 2 Catalytic Subunit (CAPN2) ELISA Kit | [8] | CSB-EL004496RA |
| InnoZyme Calpain 1/2 Activity Assay Kit | [9] | CBA054 |
| UbiQuant quantitative ubiquitin ELISA | [10] | UE101 |
| CycLex Poly-Ubiquinated Protein ELISA Kit | [11] | CY-7053 |
| 20S Proteasome Activity Assay Kit | [12] | APT280 |

[1] BioVision, Milpitas, CA, USA

[2] Alpco Diagnostics, Salem, NH, USA

[3] Cell Biolabs Inc., San Diego, CA, USA

[4] Cayman Chemical Company, Ann Arbor, MI, USA

[5] R&D Systems, Minneapolis, MN, USA

[6] Assaypro, St. Charles, MO, USA

[7] BlueGene Biotech, Shanghai, China

[8] Cusabio Biotech, Wuhan, China

[9] Calbiochem, Millipore, Schwalbach, Germany

[10] LifeSensors Inc., Malvern, PA, USA

[11] MBL International, Woburn, MA, USA

[12] Chemicon International Deutschland, Hofheim, Germany
