# Supplementary material for: Molecular Fingerprint of High Fat Diet Induced Urinary Bladder Metabolic Dysfunction in a Rat Model
Source: PLoS One. 2013 Jun 24;8(6):e66636. doi: 10.1371/journal.pone.0066636 (PMC3691244; doi:10.1371/journal.pone.0066636)
Supplement: Table S3 — High-fat diet caused massive alteration of free fatty acid profile. Plasma free fatty acids level of rats undergone chow diet (CD) and high-fat diet (HFD). Values are means ± SD for discovery set and validation set. (DOC) [file pone.0066636.s005.doc]

**Table S3:** High fat diet caused massive alteration of free fatty acid profile.

| **mean (SD)** | | **Discovery set** | | | | **Validation set** | | | |
| --- | --- | --- | --- | --- | --- | --- | --- | --- | --- |
|  | | CD n=5 | | HFD n=5 | p-value | CD n=10 | HFD n=10 | p-value | |
| **Total FFA** | | 0.71 ± 0.35 | | 1.2 ± 0.18 | ≤ 0.022 | 0.73 ± 0.25 | 1.15 ± 0.4 | ≤ 0.012 | |
| **Saturated fatty acid** | | | | | | | | | |
| **Myristic a.** | 0.01 ± 0.002 | | 0.05 ± 0.039 | | ns | 0.03 ± 0.007 | 0.04 ± 0.017 | | ns |
| **Palmitic a.** | 0.55 ± 0.36 | | 1.54 ± 0.59 | | ≤ 0.006 | 0.79 ± 0.31 | 1.18 ± 0.37 | | ≤ 0.012 |
| **Stearic a.** | 0.31 ± 0.05 | | 0.84 ± 0.26 | | ≤ 0.002 | 0.36 ± 0.11 | 0.6 ± 0.19 | | ≤ 0.004 |
| **Behenic a.** | 0.015 ± 0.003 | | 0.025 ± 0.002 | | ≤ 0.001 | 0.01 ± 0.002 | 0.02 ± 0.005 | | < 0.001 |
| **Sum** | 0.89 ± 0.08 | | 2.45 ± 0.89 | | ≤ 0.005 | 1.18 ± 0.41 | 1.81 ± 0.52 | | ≤ 0.007 |
| **Mono-unsaturated fatty acid** | | | | | | | | | |
| **Palmitoleic a.** | | 0.04 ± 0.02 | | 0.08 ± 0.05 | ns | 0.06 ± 0.02 | 0.07 ± 0.03 | | ns |
| **Oleic acid** | | 0.19 ± 0.04 | | 1.38 ± 0.62 | ≤ 0.003 | 0.49 ± 0.16 | 1.12 ± 0.35 | | < 0.001 |
| **Sum** | | 0.23 ± 0.05 | | 1.46 ± 0.66 | ≤ 0.003 | 0.56 ± 0.17 | 1.19 ± 0.36 | | < 0.001 |
| **Poly-unsaturated fatty acid** | | | | | | | | | |
| **Linoleic a.** | | 0.45 ± 0.03 | | 0.7 ± 0.33 | ns | 0.7 ± 0.32 | 0.81 ± 0.46 | | ns |
| **Arachidonic a.** | | 0.82 ± 0.12 | | 1.63 ± 0.3 | < 0.001 | 0.86 ± 0.26 | 1.35 ± 0.42 | | ≤ 0.006 |
| **α-Linolenic a.** | | 0.08 ± 0.04 | | 0.04 ± 0.02 | ns | 0.03 ± 0.01 | 0.03 ± 0.1 | | ns |
| **DHA** | | 0.08 ± 0.02 | | 0.18 ± 0.05 | < 0.002 | 0.12 ± 0.05 | 0.13 ± 0.04 | | ns |
| **Sum** | | 1.43 ± 0.12 | | 2.55 ± 0.56 | < 0.002 | 1.71 ± 0.52 | 2.32 ± 0.53 | | ≤ 0.018 |
