# Supplementary material for: Molecular Fingerprint of High Fat Diet Induced Urinary Bladder Metabolic Dysfunction in a Rat Model
Source: PLoS One. 2013 Jun 24;8(6):e66636. doi: 10.1371/journal.pone.0066636 (PMC3691244; doi:10.1371/journal.pone.0066636)
Supplement: Table S4 — Diet composition according to the data sheet information provided by ssniff Spezialdiäten GmbH, Soest, Germany. CD = chow diet; HFD = high fat diet. (DOC) [file pone.0066636.s006.doc]

**Table S4:** Macro- and micronutrient components of the diets according to the data sheet information provided by ssniff Spezialdiäten GmbH, Soest, Germany. CD = chow diet; HFD = high fat diet. Note the 11.4 times more abundance of C16:0 (palmitate) fatty acid in HFD.

| **Chow Diet (CD)** | |  | **High Fat Diet (HFD)** | |
| --- | --- | --- | --- | --- |
| metabolizable energy, calculated using Atwater-factors [1] | 15.0 MJ/kg |  | metabolizable energy, calculated using Atwater-factors [1] | 19.1 MJ/kg |
|  |  |  |  |  |
| **Minerals** | **[%]** |  | **Minerals** | **[%]** |
| calcium | 0.90 |  | calcium | 1.05 |
| phosphor | 0.63 |  | phosphor | 0.69 |
| sodium | 0.19 |  | sodium | 0.20 |
| magnesium | 0.21 |  | magnesium | 0.15 |
| potassium | 0.97 |  | potassium | 0.72 |
|  |  |  |  |  |
| **fatty acids** | **[%]** |  | **fatty acids** | **[%]** |
| C 4:0 | --- |  | C 4:0 | 0.01 |
| C 12:0 | --- |  | C 12:0 | 0.02 |
| C 14:0 | 0.02 |  | C 14:0 | 0.29 |
| C 16:0 | 0.45 |  | C 16:0 | 5.15 |
| C 16:1 | 0.02 |  | C 16:1 | 0.62 |
| C 18:0 | 0.19 |  | C 18:0 | 2.83 |
| C 18:1 | 1.07 |  | C 18:1 | 8.99 |
| C 18:2 | 2.12 |  | C 18:2 | 3.19 |
| C 18:3 | 0.26 |  | C 18:3 | 0.37 |
| C 20:0 | 0.02 |  | C 20:0 | 0.01 |
| C 20:4 | --- |  | C 20:4 | 0.35 |
| cholesterol [mg/kg] | --- |  | cholesterol [mg/kg] | 175 |
|  |  |  |  |  |
| **amino acids** | **[%]** |  | **amino acids** | **[%]** |
| lysine | 1.71 |  | lysine | 1.85 |
| methionine | 0.73 |  | methionine | 0.78 |
|  |  |  | cysteine | 0.30 |
| met+cys | 0.82 |  | met+cys | 1.08 |
| threonine | 0.93 |  | threonine | 1.00 |
| tryptophane | 0.27 |  | tryptophane | 0.29 |
| arginine | 0.76 |  | arginine | 0.82 |
| histidine | 0.66 |  | histidine | 0.71 |
| valine | 1.42 |  | valine | 1.54 |
| isoleucine | 1.09 |  | isoleucine | 1.18 |
| leucine | 2.05 |  | leucine | 2.22 |
| phenylalanine | 1.11 |  | phenylalanine | 1.21 |
| phe+tyr | 2.22 |  | phe+tyr | 2.41 |
| glycine | 0.43 |  | glycine | 0.47 |
| glutaminic acid | 4.69 |  | glutaminic acid | 5.08 |
| aspartic acid | 1.55 |  | aspartic acid | 1.68 |
| proline | 2.30 |  | proline | 2.59 |
| alanine | 0.68 |  | alanine | 0.74 |
| serine | 1.24 |  | serine | 1.34 |
|  |  |  |  |  |
| **vitamins** | **per kg** |  | **vitamins** | **per kg** |
| vitamin A | 15.000 IE |  | vitamin A | 15.000 IE |
| vitamin D_3_ | 1.500 IE |  | vitamin D_3_ | 1.500 IE |
| vitamin E | 150 mg |  | vitamin E | 150 mg |
| vitamin K | 20 mg |  | vitamin K | 20 mg |
| vitamin C | 30 mg |  | vitamin C | 30 mg |
| thiamine (B1) | 16 mg |  | thiamine (B1) | 16 mg |
| riboflavine (B2) | 16 mg |  | riboflavine (B2) | 16 mg |
| pyridoxine (B6) | 18 mg |  | pyridoxine (B6) | 18 mg |
| cobalamine (B12) | 30 µg |  | cobalamine (B12) | 30 µg |
| nicotinic acid | 49 mg |  | nicotinic acid | 45 mg |
| pantothenic acid | 56 mg |  | pantothenic acid | 55 mg |
| folic acid | 19 mg |  | folic acid | 19 mg |
| biotine | 310 µg |  | biotine | 305 µg |
| choline-Cl | 1.040 mg |  | choline-Cl | 1.050 mg |
| inositol | 80 mg |  | inositol | 80 mg |
|  |  |  |  |  |
| **micronutrients** | **per kg** |  | **micronutrients** | **per kg** |
| iron | 166 mg |  | iron | 122 mg |
| manganese | 98 mg |  | manganese | 72 mg |
| zinc | 65 mg |  | zinc | 50 mg |
| copper | 14 mg |  | copper | 10 mg |
| iodine | 1.2 mg |  | iodine | 0.85 mg |
| selenium | 0.14 mg |  | selenium | 0.12 mg |
| cobalt | 0.15 mg |  | cobalt | 0.11 mg |

[1] Southgate DAT The relationship between food composition and available energy. Joint FAO/WHO/UNU Expert Consultation on Energy and Protein Requirements, Rome, 5 to 17 October 1981. URL: http://www.fao.org/docrep/MEETING/004/M2847E/M2847E00.HTM Retrieved 2013-04-11.
